# King Edward's Hospital Fund for London

**Published:** 1913-01-18

**Authors:** 


### King Edward's Hospital Fund for London.

A meeting of the General Council of King Edward's Hospital Fund for London was held last Monday afternoon at the Offices of the Fund, 7 Walbrook, E.C.

There were present Sir W. S. Church, Bart (in the chair), the Right Hon. Sir Savile Crossley, Bart. (Hon. Secretary), Sir Horace Marshall, Sir William J. Collins, Sir Cooper Parry, Sir John Craggs, Sir John Tweedy, Dr. Edwin Freshfield, Mr. Robert Fleming, Mr. John G. Griffiths (Hon. Secretary), Mr. Albert G. Sandeman.

Sir Savile Crossley (Hon. Secretary) stated that the Governors had appointed the following new members of the General Council: The Rev. W. L. Watkinson, D.D., in the place of the late Rev. T. Bowman Stephenson, D.D.; the Right Hon. Sir T. Vezey Strong, Sir John Tweedy, Mr. Robert Fleming, and Mr. A. E. Sydney.

The order of appointment, signed by the Governors, of the members of the General Council, members of committees, and officers for the year 1913 was read. The resolutions providing for the work of the Fund for 1913, which were approved at the meeting of the Governors and General Council held at St. James's Palace on December 18, 1912, were formally adopted.

